# Mast cell involvement in glucose tolerance impairment caused by chronic mild stress with sleep disturbance

**DOI:** 10.1038/s41598-017-14162-w

**Published:** 2017-10-20

**Authors:** Sachiko Chikahisa, Saki Harada, Noriyuki Shimizu, Tetsuya Shiuchi, Airi Otsuka, Seiji Nishino, Hiroyoshi Séi

**Affiliations:** 1 Department of Integrative Physiology, Institute of Biomedical Sciences, Tokushima University Graduate School, Tokushima, 770-8503 Japan; 2 Student lab, Tokushima University Faculty of Medicine, Tokushima, 770-8503 Japan; 3 Sleep & Circadian Neurobiology Laboratory, Stanford University School of Medicine, Palo Alto, CA United States

## Abstract

We have developed a chronic mild stress (MS) mouse model by simply rearing mice on a wire net for 3 weeks and investigated the effects of MS on glucose homeostasis and sleep. MS mice showed impaired glucose tolerance and disturbed sleep. One-week treatment with a histamine H1 receptor antagonist (H1RA) ameliorated the glucose intolerance and improved sleep quality in MS mice. MS mice showed an increased number of mast cells in both adipose tissue and the brain. Inhibition of mast cell function ameliorated the impairment in both glucose tolerance and sleep. Together, these findings indicate that mast cells may represent an important pathophysiological mediator in sleep and energy homeostasis.

## Introduction

We recently found that mast cells in the brain play a significant role in the regulation of sleep and fundamental neurobehaviour such as food-seeking behaviour^1^. Mast cells are immune cells derived from bone marrow progenitors^2–4^. These cells infiltrate most tissues, mature in local tissues depending on microenvironmental factors and then produce and secrete numerous mediators including histamine^2,3^. In addition to their role in allergic inflammation and host defence to immunologic stimuli in peripheral tissues, mast cells infiltrate and reside in the brain, especially in the thalamus, hypothalamus, hippocampus, and the leptomeninges overlying these areas^5–8^. Numerous data indicate that the activation and population of mast cells in the brain are altered in response to a wide variety of environmental stimuli, including stress. For example, the brain population of mast cells is increased by chronic subordination stress, such as exposure to a fighting opponent in mice^9^, while handling or immobilization stress reduces the total number of brain mast cells in rats^10^. Recent studies have also established that mast cells in the white adipose tissue (WAT) contribute to diet-induced obesity and diabetes in both humans^11,12^ and mice^13,14^.

In the present study, we therefore developed a chronic mild stress (MS) model that is useful in the evaluation of chronic hypnotic effects by simply placing mice on a wire net. Using this model, we studied the effects of chronic environmental stress on energy metabolism and sleep homeostasis. Our results indicate that MS induced notable changes in glucose and sleep homeostasis with an increased number of mast cells in the brain and adipose tissue.

## Results

### Rearing on a wire net for 3 weeks impaired glucose tolerance

**Figure 1:**
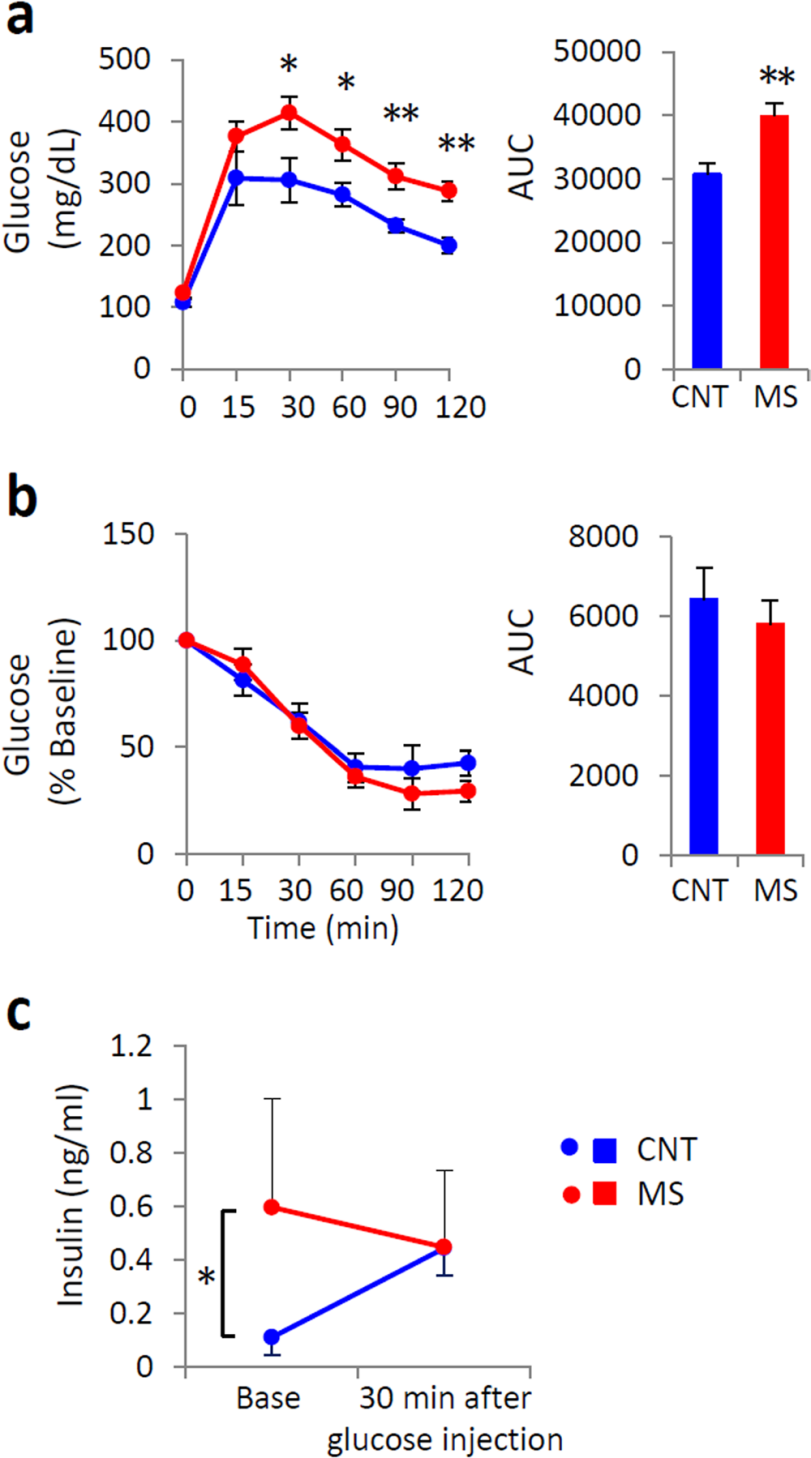
Chronic mild stress (MS) impaired glucose tolerance. An intraperitoneal glucose tolerance test (GTT) (**a**) and an insulin tolerance test (ITT) (**b**) were performed in control (CNT) and MS mice via rearing on a wire net for 3 weeks. The area under the blood glucose curve (AUC) from GTT and ITT measurements is shown in the right panels. Plasma insulin levels in response to an intraperitoneal glucose injection after 3 weeks of MS (**c**). Blue circles and bars indicate CNT mice, and red circles and bars indicate MS mice. All data are expressed as the means ± SEM (**a**,**b**: n = 8–11/group, **c**: n = 5/group). *p < 0.05, **p < 0.01, CNT versus MS mice.

**Figure 2:**
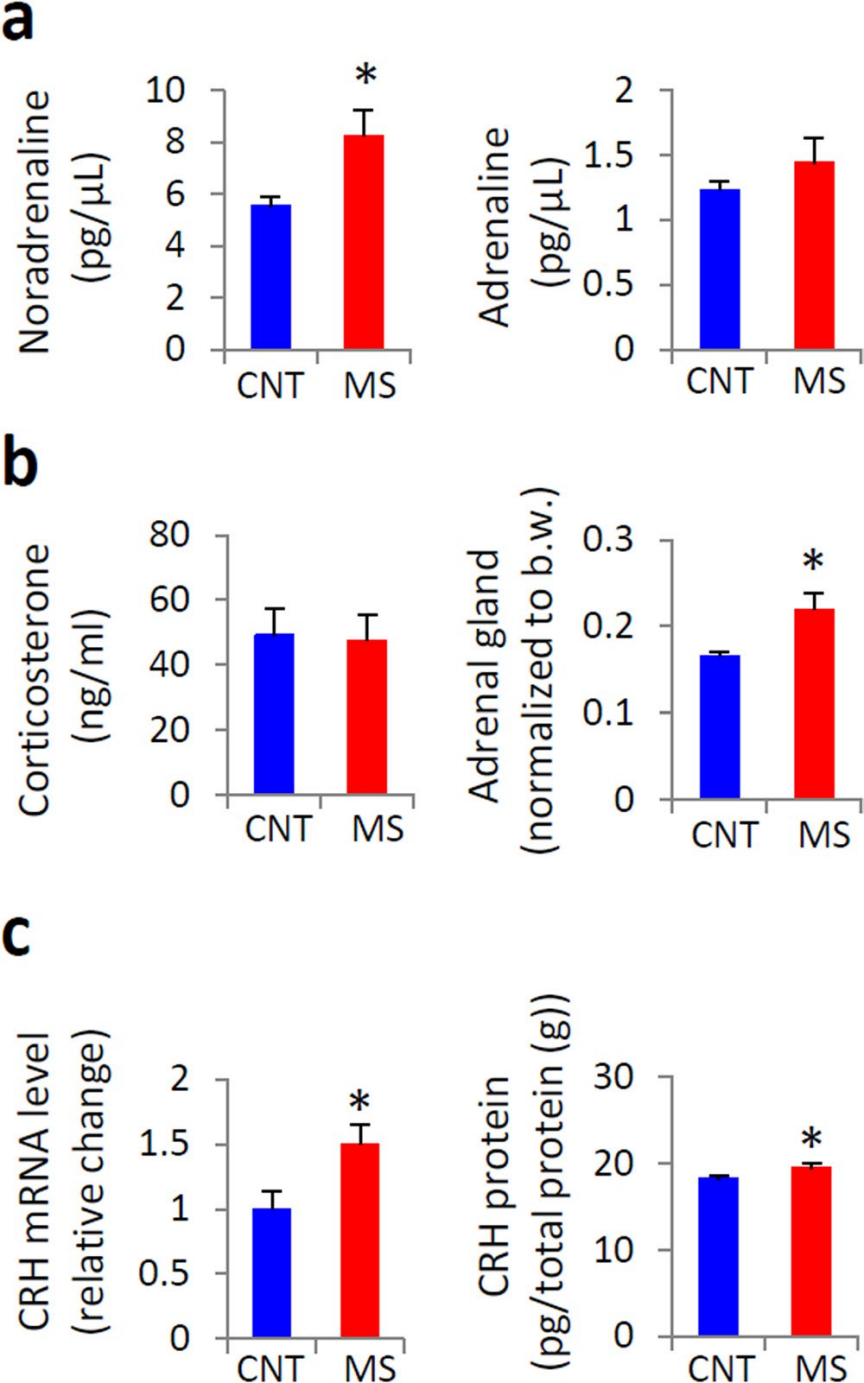
Effects of rearing on a wire net for 3 weeks on the stress response. Plasma noradrenaline and adrenaline levels (**a**), plasma corticosterone levels and adrenal gland weights (**b**), and mRNA and protein expression of corticotropin-releasing hormone (CRH) in the hypothalamus (**c**) were measured. Blue bars indicate control (CNT) mice, and red bars indicate chronic mild stress (MS) mice. All data are expressed as the means ± SEM (n = 7–11/group). *p < 0.05, CNT versus MS mice.

MS mice reared on a wire net for 3 weeks had significantly higher glucose levels at 30, 60, 90, and 120 min in the glucose tolerance test (GTT) (Fig. 1a). The insulin tolerance test (ITT) results were not altered in MS mice (Fig. 1b). However, the plasma insulin response to glucose was disrupted in MS mice (Fig. 1c). In MS mice, plasma noradrenaline levels were significantly higher than in control (CNT) mice (Fig. 2a). The expression of corticotropin-releasing hormone (CRH) mRNA and protein in the hypothalamus and the adrenal gland weight of MS mice were also increased, although the plasma concentration of corticosterone was at a similar level to that of CNT (Fig. 2b,c). These results suggest that rearing on a wire net for 3 weeks resulted in stress responses with a small but significant activation of the HPA axis and/or sympathetic nervous system. However, there was no significant difference in body weight, food intake, O_2_ consumption (VO_2_), CO_2_ production (VCO_2_), the respiratory quotient (RQ), or the plasma concentration of glucose, triglycerides, free fatty acids, cholesterol, or ketone bodies between CNT and MS mice (Supplementary Fig. S1a–d).

### Rearing on a wire net for 3 weeks also disturbed sleep

**Figure 3:**
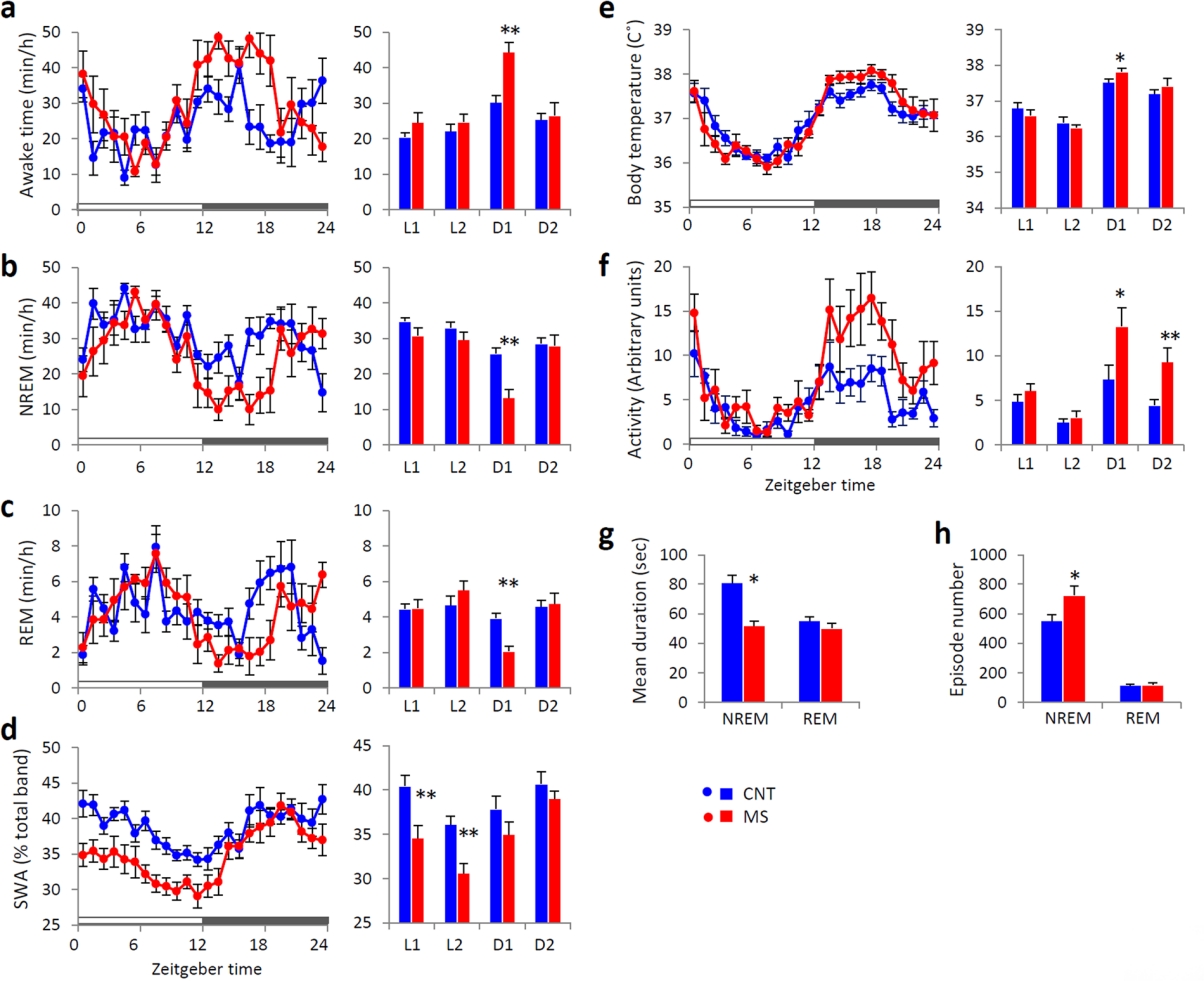
Effects of chronic mild stress (MS) on sleep in mice. Hourly time course (left panels) and 6-hour bins (right panels; ZT0-6 (L1), ZT6-12 (L2), ZT12-18 (D1) and ZT18-24 (D2)) for wakefulness (**a**), non-rapid eye movement (NREM) sleep (**b**), rapid eye movement (REM) sleep (**c**), slow-wave activity (SWA) in NREM sleep (**d**), body temperature (**e**), and locomotor activity (**f**). Mean duration of bouts (**g**) and episode number (**h**) of NREM and REM sleep across 24 hours. Blue circles and bars indicate control (CNT) mice, and red circles and bars indicate MS mice. All data are expressed as the means ± SEM (n = 7/group). *p < 0.05, **p < 0.01, CNT versus MS mice.

MS mice showed increased wakefulness, decreased amount of non-rapid eye movement (NREM) and rapid eye movement (REM) sleep in the first half of the dark phase, and attenuated slow-wave activity (SWA) during NREM sleep in the light phase compared with those in CNT mice (Fig. 3a–d). MS mice showed a decreased mean bout duration of NREM sleep and increased episode number of NREM sleep, suggesting that sleep in MS mice demonstrated increased fragmentation (Fig. 3g and h). The body temperature and locomotor activity of MS mice were also increased in the dark phase, the period when wakefulness was increased (Fig. 3e and f).

To investigate the timing of the sleep impairment and impaired glucose tolerance, we evaluated sleep and GTT in mice reared on a wire net for 1 week. Rearing on a wire net for 1 week disturbed sleep but did not impair glucose tolerance (Supplementary Fig. S2a–h). These data indicate that sleep disturbance is followed by impaired glucose tolerance.

### Two classes of sleep-inducing agents had different effects on GTT and sleep

**Figure 4:**
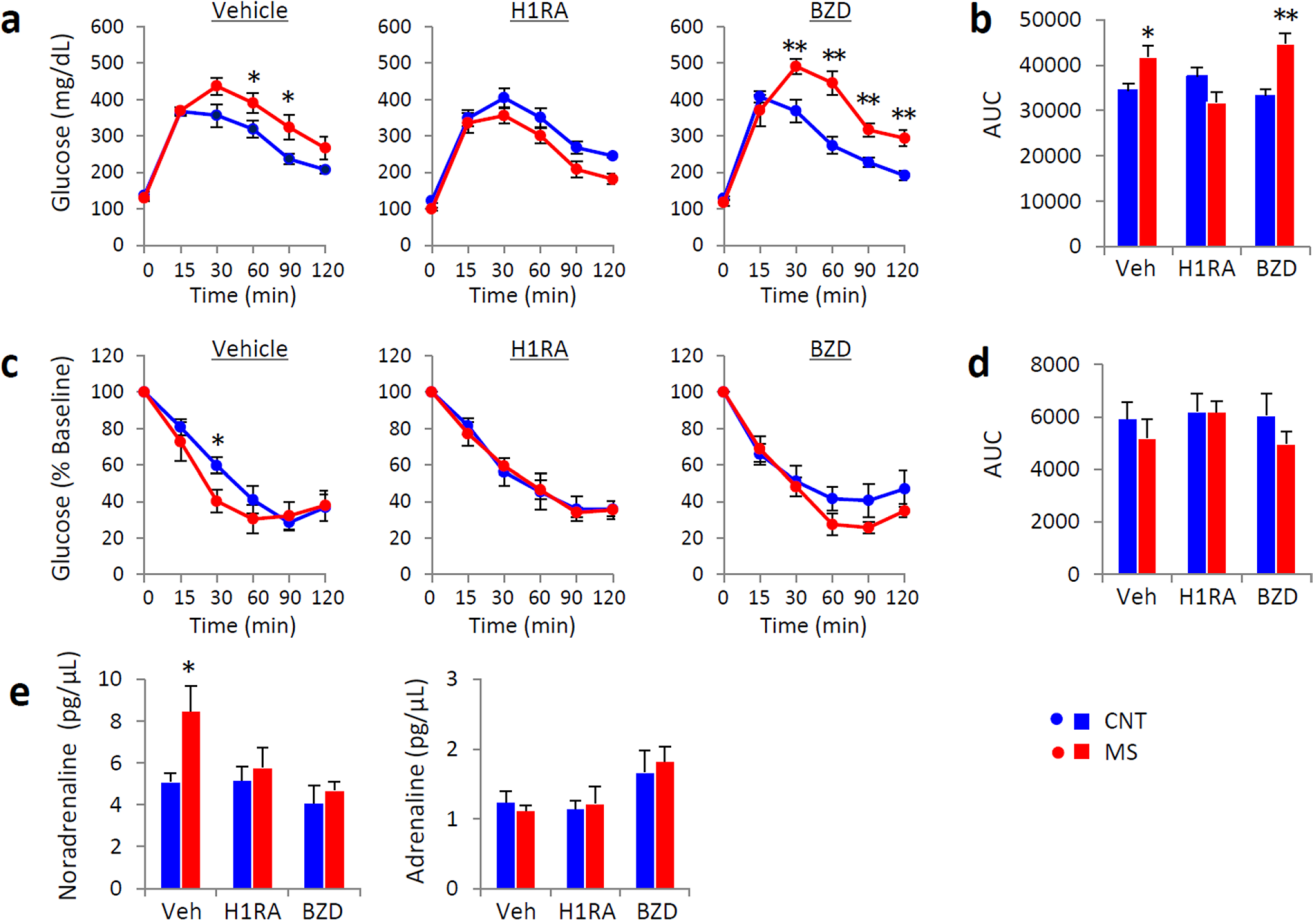
Two kinds of sleep-inducing agents had differential effects on glucose tolerance in control (CNT) and chronic mild stress (MS) mice. An intraperitoneal glucose tolerance test (GTT) (**a**) and an insulin tolerance test (ITT) (**c**) were performed in mice injected with vehicle (left panels), a histamine H1 receptor antagonist (H1RA, 5 mg pyrilamine/kg body weight, i.p.) (middle panels), or a benzodiazepine (BZD, 2 mg diazepam/kg body weight, i.p.) (right panels). The area under the blood glucose curve (AUC) for GTT (**b**) and ITT (**d**) values is shown in the right panels. Plasma noradrenaline and adrenaline levels (**e**) were measured in MS mice injected with each drug. Blue circles and bars indicate CNT mice, and red circles and bars indicate MS mice. All data are expressed as the means ± SEM (n = 6/group). *p < 0.05, **p < 0.01, CNT versus MS mice.

We next evaluated the effect of sleep-inducing agents on sleep and glucose tolerance in a different group of MS mice by one-week treatment with both a histamine H1 receptor antagonist (H1RA) and benzodiazepine (BZD), a commonly used class of hypnotic sleep-inducing agents in humans. Each drug was administered to mice over the last 1 week of rearing on the wire net for 3 weeks. The GTT values of H1RA-treated MS mice did not differ from those of CNT mice, while the BZD-treated MS mice still had higher glucose levels than BZD-treated CNT mice (Fig. 4a and b). There was no significant difference in ITT values between the CNT and MS mice injected with drugs (Fig. 4c and d). Increased plasma noradrenaline levels observed in vehicle-treated MS mice were suppressed by treatment with both H1RA and BZD (Fig. 4e), indicating that both types of hypnotic drugs could suppress the hyperactivity of sympathetic tone by MS.

**Figure 5:**
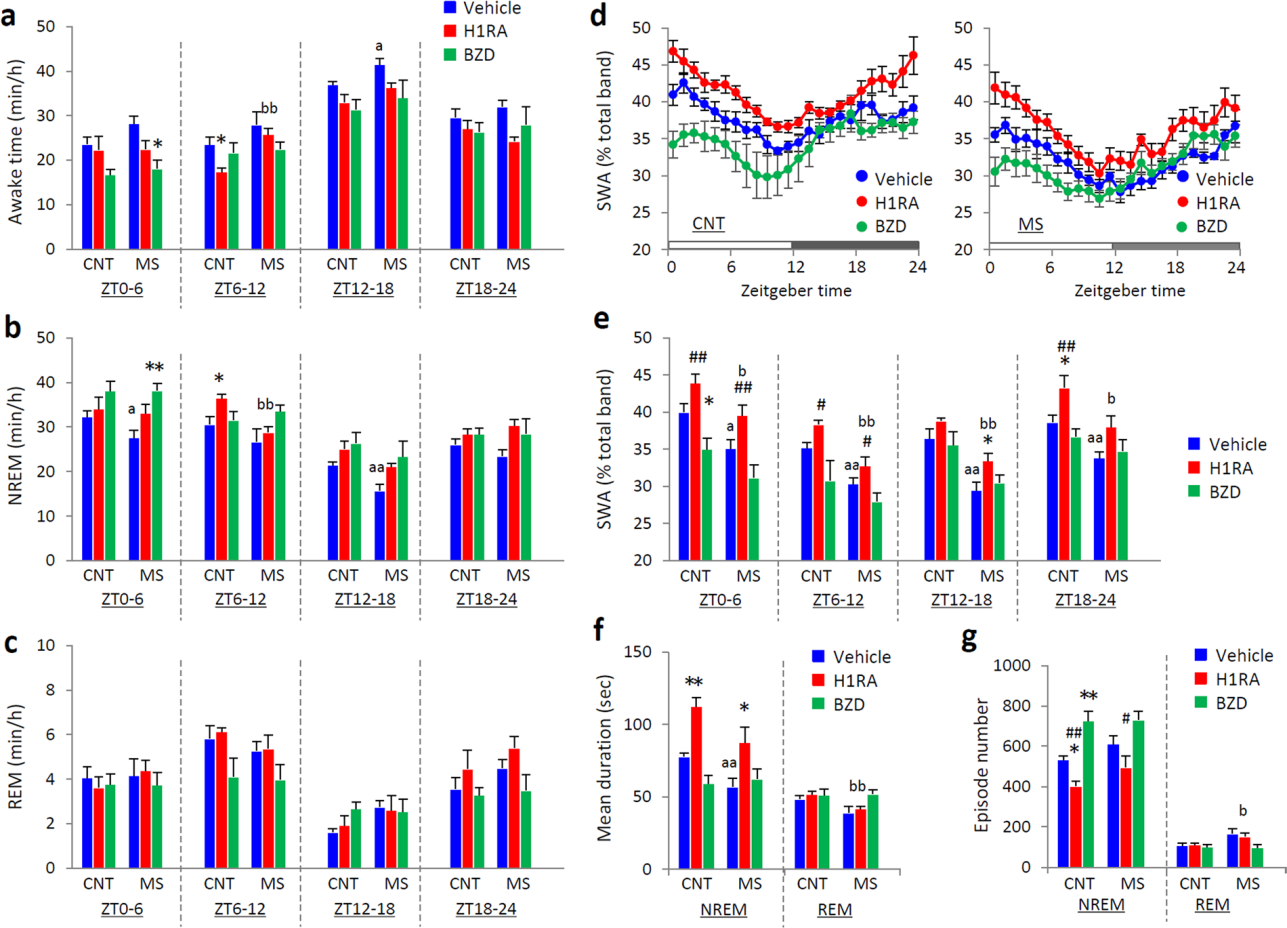
Two kinds of sleep-inducing agents exhibited differential effects on sleep in control (CNT) and chronic mild stress (MS) mice. Time course for 6-hour bins (ZT0-6, ZT6-12, ZT12-18 and ZT18-24) of wakefulness (**a**), non-rapid eye movement (NREM) sleep (**b**), rapid eye movement (REM) sleep (**c**), and slow-wave activity (SWA) in NREM sleep (**e**) in mice injected with vehicle, histamine H1 receptor antagonist (H1RA), or benzodiazepine (BZD). Hourly time course for SWA during NREM sleep (**d**) and mean duration of bouts (**f**)/episode number (**g**) of NREM and REM sleep across 24 hours after injection of each drug. Blue circles and bars indicate mice injected with vehicle, red circles and bars indicate H1RA administration (5 mg pyrilamine/kg body weight, i.p.), and green circles indicate BZD administration (2 mg diazepam/kg body weight, i.p.). All data are expressed as the means ± SEM (n = 6/group). *p < 0.05, **p < 0.01, versus vehicle; ^#^p < 0.05, ^##^p < 0.01, versus BZD. ^a^p < 0.05, ^aa^p < 0.01, vehicle-treated CNT versus vehicle-treated MS; ^b^p < 0.05, ^bb^p < 0.01, H1RA-treated CNT versus H1RA-treated MS.

Regarding sleep, just as seen in the baseline recording (Fig. 3), vehicle-treated MS mice showed the increased wakefulness, decreased NREM sleep and suppressed SWA compared with vehicle-treated CNT mice (Supplementary Fig. S3a,b and d). One-week treatment with both a H1RA and BZD decreased the amount of wakefulness and increased the amount of NREM sleep in both CNT and MS mice (Fig. 5a and b). Treatment with H1RA enhanced SWA during NREM sleep in both groups, while BZD decreased it (Fig. 5d and e). The SWA in H1RA-treated MS mice recovered to the level of that in vehicle-treated CNT mice, while that in BZD-treated MS mice was significantly decreased compared with that in vehicle-treated CNT mice (Supplementary Fig. S3d). Similarly, only the mice treated with H1RA displayed decreased sleep fragmentation in both CNT and MS mice (Fig. 5f and g). Neither H1RA nor BZD affected body weight, food intake or the plasma concentration of glucose, triglycerides, free fatty acids, or cholesterol in both CNT and MS mice (Supplementary Fig. S4a–c). These data suggest that H1RA treatment ameliorated the impaired GTT and sleep observed in MS mice, and histamine-related function is associated with the stress-induced GTT and sleep impairment.

### Sleep disturbance increased the number of mast cells in the WAT and brain

**Figure 6:**
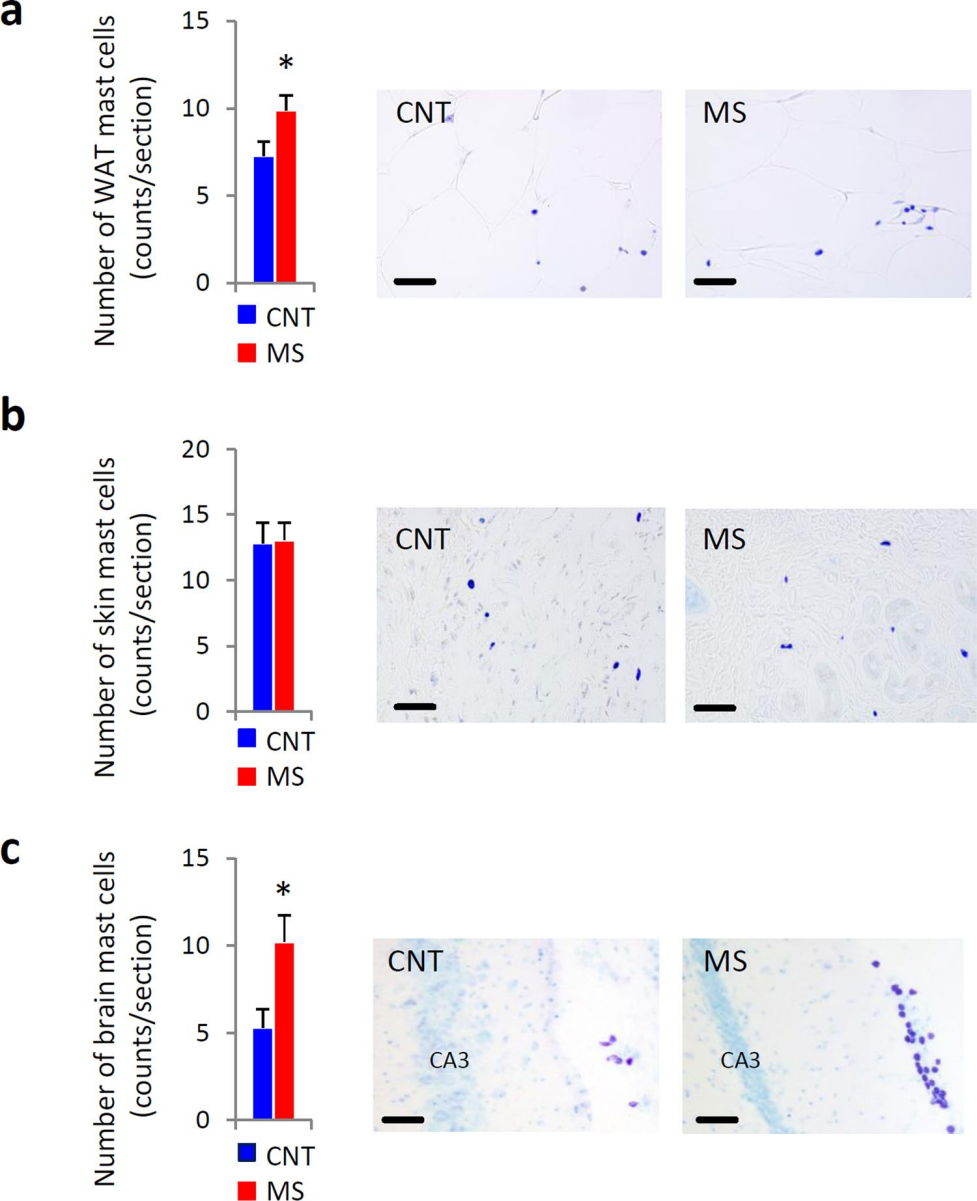
Chronic mild stress (MS) for 3 weeks increased the number of mast cells. Toluidine blue staining detected mast cell numbers in the white adipose tissue (WAT) (**a**), volar skin of the lower legs (**b**), and brain (**c**) from control (CNT) and MS mice. Scale bar, 50 μm (**a** and **b**) and 100 μm (**c**). Blue bars indicate CNT mice, and red bars indicate MS mice. All data are expressed as the means ± SEM (**a**,**b**: n = 6/group, c: n = 13/group). *p < 0.05, versus CNT. Abbreviations: field CA3 hippocampus (CA3).

We next counted mast cell numbers in the WAT, skin and brain. WAT from MS mice contained more mast cells than that from CNT mice, while there was no difference in mast cell number in the volar skin of the lower legs from CNT and MS mice (Fig. 6a and b). Brains from MS mice also contained more mast cells than CNT mice, especially in the periventricular organs around the hippocampus and thalamus (Fig. 6c).

Basal histamine contents (cortex and thalamus) and release (measured in the lateral ventricle via microdialysis) were higher than the levels in CNT mice (Supplementary Fig. S5a). However, the expression of histidine decarboxylase (HDC) mRNA in the hypothalamus was not different between the two groups (Supplementary Fig. S5b). Although immunofluorescent double-staining showed higher *c-fos* expression in HDC-positive tuberomammillary nucleus (TMN) cells from orexin B-injected mice (positive control), *c-fos* expression in the histamine neurons of MS mice was not significantly higher than in CNT mice (Supplementary Fig. S5c). These results suggest that an increase in histamine levels observed in MS mice is largely due to brain-resident mast cells, rather than TMN activity.

### Mast cell deficiency prevented disruption of GTT values

**Figure 7:**
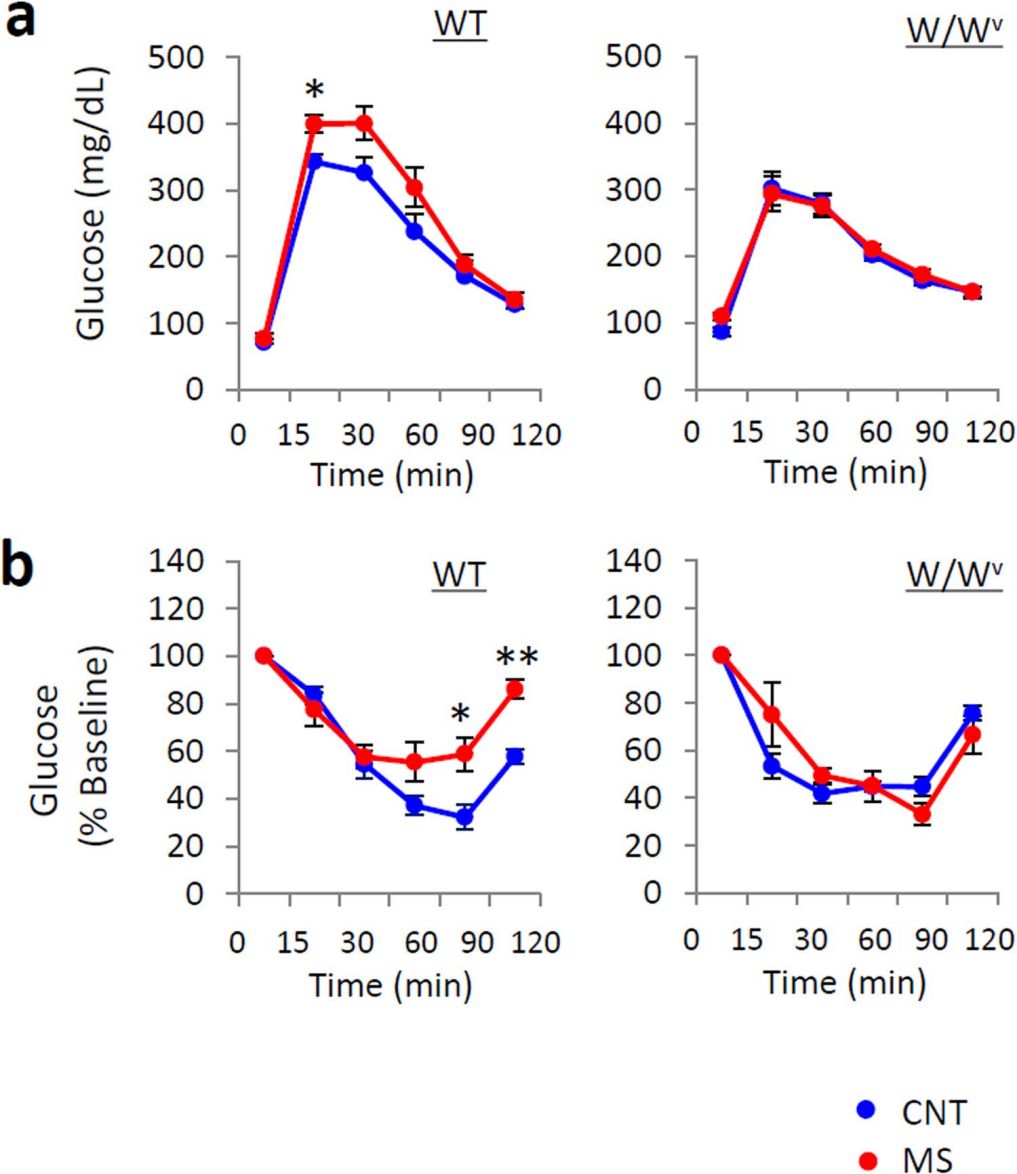
Deletion of mast cells ameliorated the impairment of glucose tolerance induced by chronic mild stress (MS). Glucose tolerance test (GTT) (**a**) and an insulin tolerance test (ITT) (**b**) were performed in wild-type (WT) and mast cell-deficient (W/W^v^) mice. Blue circles indicate control (CNT) mice, and red circles indicate MS mice. All data are expressed as the means ± SEM (n = 5–6/group). *p < 0.05, versus CNT.

To examine the contribution of mast cells to glucose tolerance, we evaluated GTT measures in wild-type (WT) and mast cell-deficient *Kit* ^*W/Wv*^ (W/W^v^) mice after rearing on control bedding or wire nets for 3 weeks. WT mice reared on a wire net for 3 weeks exhibited worsened GTT values, although W/W^v^ mice reared on a wire net showed no change in their GTT measures (Fig. 7a). These results suggest that mast cells are essential for the stress-induced glucose impairment observed in MS mice.

## Discussion

This study shows that MS by rearing on a wire net impairs glucose and sleep homeostasis, which is accompanied by an increased population of mast cells in the WAT and brain. Treatment with H1RA or suppression of mast cell function ameliorated the GTT measures and/or sleep quality deficits that were impaired in MS mice. These data suggest that MS impairs glucose and sleep homeostasis and that mast cells may represent a critical mediator in their pathophysiological mechanism.

In the present study, mice reared on a wire net for 3 weeks showed an attenuated SWA during NREM sleep and increased sleep fragmentation, accompanied by an impaired glucose tolerance. The elevated glucose levels that occurred after a 3-week rearing on a wire net were not observed after a 1-week sleep disturbance, suggesting that chronic sleep disruption may precede the impairment of glucose tolerance. Although both types of hypnotic drugs could suppress the serum noradrenalin, BZD did not improve the GTT in MS mice. Therefore, the impairment of GTT in MS mice was considered to not only be caused by noradrenaline, that is sympathetic hyperactivity.

Both the impaired GTT and sleep observed in MS mice were improved by treatment with pyrilamine, a selective and high-affinity H1RA. Mast cells store approximately 50% of the histamine content in the brain, since brain histamine levels in mast cell-deficient mice are approximately 50% of that in wild-type mice^15^. We recently reported that histamine released from brain mast cells is wake-promoting^1^. Brain resident mast cells can be reasonably assumed to be increased or activated in MS mice, which leads to increased histamine levels in the brain associated with sleep disturbance. Actually, the amount of SWA can be ordered as follows: CNT with H1R antagonist > CNT with vehicle ≒ MS with H1R antagonist > MS with vehicle (Fig. 5d and e). On the other hand, as SWA response against H1RA is similar in both group, H1RA efficacy itself is not considered to be affected by MS.

Brain mast cells may mediate the changes in both sleep and glucose homeostasis, since 1-week intracerebroventricular (i.c.v.) injection of the mast cell degranulation blocker cromolyn ameliorated the impaired sleep quality and the deterioration of GTT values in MS mice (Supplementary Figs S6 and S7). The sleep data in the cromolyn experiment seems to be inconsistent with other baseline and pharmacological (H1RA/BZD) experiments. Although significant increase in sleep fragmentation and tendency of suppression in SWA were seen in vehicle-treated MS mice, there were no significant differences in the amount of sleep between vehicle-treated CNT and vehicle-treated MS mice (Supplementary Fig. S7). GTT response was also relatively small. In the cromolyn experiment, chronic manipulations of i.c.v. drug injection for 1 week may have some influence on sleep amount, similar to that of another stressor, even in mice treated with vehicle. Even so, in also this cromolyn experiment, sleep fragmentation and impaired GTT were seen in the vehicle-treated MS mice. These facts suggest that rearing on a wire net mainly affects sleep quality but not its amount. And, the both sleep fragmentation and impaired GTT were recovered by cromolyn. Thus, sleep quality may be more critical than sleep amount for the regulation of glucose homeostasis, as reported in previous human studies^16,17^.

MS mice also showed an increased number of mast cells in WAT. Therefore, these mast cells may mediate the changes in glucose homeostasis. A recent study has reported that the number of mast cells in the WAT of obese mice was higher than in that of lean counterparts^14^. In addition, mast cell-deficient (*Kit* ^*W−sh/W−sh*^) mice or WT mice receiving a mast cell stabilizer showed a lower rate of diet-induced obesity and diabetes than WT control mice^14^. A mast cell stabilizer also reduces fasting blood glucose, HbA1c, low-density lipoproteins, and triglycerides in diabetic patients^12^. These results are in agreement with our results that MS resulted in normal glucose tolerance of mast cell-deficient mice. H1RA treatment may also normalize glucose tolerance through peripheral mechanisms, since chronic treatment with H1RA (cetirizine, a second generation H1RA with low central penetration) has been recently reported to ameliorate glucose intolerance in high-fat diet mice^18^. Histamine released from peripheral mast cells may not be able to affect sleep because peripheral histamine does not cross the blood–brain barrier. In a future study, the relationship between central and peripheral mast cell function should be further clarified.

Mast cells are known to express CRH receptors and can be stimulated by CRH under stress^19,20^. CRH is a neuropeptide that is essential for the stress response. In the present study, the expression of CRH mRNA and protein in the hypothalamus and adrenal gland weight were increased in MS mice, although the plasma corticosterone level was not changed. Moreover, sympathetic nerve activity in MS mice would also be chronically enhanced, since plasma noradrenaline levels are thought to closely reflect the activity of sympathetic nerves^21^. MS mice would likely exhibit stress-induced enhancement of CRH levels that would be expected to stimulate mast cells, leading to impairment of sleep and glucose homeostasis.

In conclusion, MS by rearing on a wire net induces an impairment of glucose and sleep homeostasis, accompanied by increased plasma noradrenaline and CRH in the hypothalamus. We identified increased brain histamine levels and number of mast cells in the WAT and brain as one likely mechanism underlying this response by showing that treatment with an H1RA improved glucose tolerance and sleep quality induced by MS, and mast cell dilation prevented disruption of glucose tolerance (Supplementary Fig. S7). Our findings demonstrate new mechanisms for chronic sleep disturbance and glucose homeostasis abnormalities, and these may lead to new recognition of the potential pathophysiological roles of mast cells.

## Methods

### Animals

Adult male Jcl/ICR mice (Slc Inc., Shizuoka, Japan) were used in most experiments. Only in the case of the experiment using mast cell-deficient mice (Fig. 7), W/W^v^ (WBB6F1/Kit-*Kit* ^*W*^ */Kit* ^*W-v*^/Slc, Slc Inc., Shizuoka, Japan) and their wild-type were used. Mice were fed *ad libitum* and maintained on a 12-hour light-dark (L/D) cycle (lights on at 0900) at a controlled ambient temperature (24 ± 1 °C). Mice (aged 8 weeks old) were housed individually in separate cages (18 × 26 × 13 cm) and randomly assigned to two groups to be reared on either normal sawdust (control) or wire mesh (chronic mild stress, MS). Wire mesh was made of a stainless grid, and the square size of each grid was 6 mm. These experiments were performed with the approval of The Animal Study Committee of Tokushima University, and we performed them in accordance with Guidelines for the Care and Use of Animals approved by the Council of the Physiological Society of Japan.

### Sleep recording and analysis

Electroencephalogram (EEG)/electromyogram (EMG) implantation surgery for sleep recording and telemetry (TA10TA-F20; Data Sciences Int., USA) for recording of body temperature and locomotor activity were performed as previously described^22^. Off-line sleep scoring was done on the computer screen by visual assessment of EEG and EMG activity using the Spike2 analysis program (CED, Cambridge, UK). Vigilance states were based on data binned in 6-second epochs and classified as wakefulness, REM or NREM sleep. The EEG power spectrum in the epoch determined to represent NREM sleep was calculated by Fast Fourier Transform using the Spike2 analysis program. The slow-wave activity (SWA; EEG delta frequency band) was set at 0.5–4.0 Hz, and the delta power was normalized as a percentage of the total power (0.5–50 Hz). Body temperature, locomotor activity, time spent sleeping and awake, and SWA were averaged for hourly intervals.

### GTT and ITT

Mice were food-deprived overnight for 18 hours (GTT) or for 5 hours (ITT). At *Zeitgeber* Time (ZT) 4, a blood sample was taken via a small tail nick, and the fasted baseline blood glucose level was determined by a glucose biosensor (LifeScan, Inc., Milpitas, CA, USA). Blood samples were collected at 15, 30, 60, 90, and 120 minutes after intraperitoneal injection (i.p.) of glucose (2 g/kg) or insulin (1 U/kg).

### Pharmacological treatments and injection procedures

In the pharmacological experiments, we prepared different groups of MS and CNT mice. Each drug was administered to mice over the last 1 week of rearing on the wire net for 3 weeks. A histamine H1 receptor antagonist (H1RA) (pyrilamine, 5 mg/kg, i.p.) or benzodiazepine (BZD)(diazepam, 2 mg/kg, i.p.) was administered to each animal at ZT23.5. These drug injections during the dark period were done under a dim, red light. A mast cell stabilizer (cromolyn, 30 μg, i.c.v.) was injected slowly over the course of 1 minute, using a Hamilton microsyringe at ZT0 over 6 consecutive days. Cannulae were implanted intracerebroventricularly at the time of surgery for EEG/EMG, as previously described^23^. Sleep data for the 24 hours following the last injection were captured and analysed.

### Real-time RT-PCR analysis

Tissues used for molecular analysis were dissected immediately after decapitation at ZT12, frozen in liquid nitrogen, and stored at −80 °C until use. Tissue preparation and analysis were performed as previously described^22^. We used pre-designed, gene-specific TaqMan probes and primer sets (Applied Biosystems, Foster City, CA) to assess the expression of the following genes: *HDC* (Mm00456104_m1) and *CRH* (Mm01293920_s1). Real-time RT-PCR was carried out using an Applied Biosystems StepOnePlus and TaqMan universal PCR Master Mix (Roche Applied Science, Mannheim, Germany) according to the manufacturer’s instructions. For endogenous quantity control, we normalized values to those for the housekeeping gene *β-actin* (Mm00607939_s1).

### CRH enzyme-linked immunosorbent assay (ELISA)

Frozen samples were diluted with RIPA Buffer (Nakarai Tesque, Kyoto, Japan). CRH protein was assessed using an ELISA kit following the vendor’s instructions (YK131, Yanaihara, Shizuoka, Japan). The absorbance was read using a plate reader adjusted to 450 nm. Total protein in each tissue sample was assessed using a BioRad protein assay. The proportion of CRH/mg total protein for each sample was then calculated.

### Double-label immunofluorescence protocol

Mice were anaesthetized with a cocktail of ketamine (100 mg/kg) and xylazine (25 mg/kg, i.p.) and perfused with 60 ml saline, followed by 70 ml 4% paraformaldehyde in 0.1 M phosphate buffer (PB). The brains were removed and immersed in the same fixative for 24 hours at 4 °C, and the solution was replaced with PB containing 30% sucrose and 0.1% sodium azide for 48 hours. Then, the brains were frozen in O.C.T. compound (Sakura Finetechnical Co. Ltd., Tokyo, Japan). The brains were coronally sectioned (30 μm thickness) on a freezing cryostat. For HDC and c-fos double-staining, sections were incubated with a goat anti-HDC antibody (Santa Cruz Biotechnology, Santa Cruz, CA) and rabbit anti-c-fos antibody (Cell Signaling Technology, Beverly, MA) diluted 1:500 in PB saline containing 0.3% Triton X-100 (PBS-T) and 0.1% sodium azide for 72 hours at 4 °C. These sections were incubated with the Alexa 594-labelled donkey anti-goat IgG antibody or Alexa 488-labelled donkey anti-rabbit IgG (1:500, Life Technologies, Gaithersburg, MD) for 2 hours at room temperature. The sections were mounted on glass slides and examined with a fluorescence microscope (DM4000, DFC550, Leica Japan, Tokyo, Japan).

### Measurement of plasma levels of triglycerides, free fatty acids (FFAs), ketone bodies, and glucose

Trunk blood was collected for measurement of triglyceride, cholesterol, FFA, and ketone body (acetoacetate and β-hydroxybutyrate) levels. Plasma triglyceride, cholesterol, and FFA levels were determined by GPO-HDAOS (triglycerides), HMMPS (cholesterol), ACS-ACOD (FFAs), and glucose (HK-UV) enzyme assays using an automatic biochemical analyser system (HITACHI 7180, Hitachi, Tokyo, Japan and JCA-DM2250, JEOL, Tokyo, Japan). Ketone bodies were measured by an automatic analyser system JCA-BM12 (JEOL, Tokyo, Japan) using reagents for measurement of ketone bodies by enzymatic assay (Kainos Laboratories, Tokyo, Japan).

### Measurement of plasma levels of corticosterone and insulin

Trunk blood for corticosterone measurement was collected from each group at the time of decapitation for real-time RT-PCR analysis. The plasma corticosterone level was determined using an enzyme immunoassay kit (Yanaihara Institute Inc., Fujinomiya, Japan). The blood samples for insulin measurements were obtained from the orbital venous plexus using capillary glass tubes at the 18-hour fasted baseline and 30 min after glucose injection (2 g/kg, i.p.). The blood insulin levels were determined by an insulin ELISA kit (Shibayagi, Shibukawa, Japan).

### Plasma noradrenaline and adrenaline analysis

Mice were not injected with any drugs on the day of sampling. The plasma concentration of noradrenaline and adrenaline was determined using an Eicom high-performance liquid chromatography-electrochemical detector (HPLC-ECD) system. Purified plasma samples by adsorption on alumina were injected onto a column (Eicompak SC-5ODS, 150 mm × 3.0 mm i.d.) with a pre-column (CA-ODS, 4 mm × 3.0 mm i.d.). Noradrenaline and adrenaline were eluted with 0.1 M sodium phosphate buffer at a flow rate of 0.23 ml/min and detected with an ECD-300 detector (Eicom, Kyoto, Japan).

### *In vivo* microdialysis

The microdialysis cannula was implanted into the left lateral ventricle (AP -0.5 mm; ML 1.2 mm; V 1.5 mm to bregma), and the cannula for drug injection was implanted obliquely into the right lateral ventricle (AP -2.2 mm; ML 0.9 mm; V 2.5 mm, Angle 30° relative to bregma) of mice under general anaesthesia. A microdialysis probe with a 2-mm-long semipermeable membrane (Eicom, Kyoto, Japan) was inserted into the lateral ventricle 3 to 4 hours prior to the experiment. The microdialysis lines were continuously perfused with Ringer’s solution at a rate of 1 μl/min. Histamine content in the cerebrospinal fluid was determined by using the HPLC-fluorometry system following the vendor’s instructions (Eicom, Kyoto, Japan). Dialysate samples (30 μl) were injected directly onto a column (Eicompak SC-5ODS, 150 mm × 3.0 mm i.d.). The histamine was eluted with 0.25 M potassium phosphate at a flow rate of 0.5 ml/min, was post-labelled with o-phthalaldehyde in an alkaline condition and was then detected fluorometrically in a Fluorometer (FL7753, GL Sciences, Tokyo, Japan).

### Statistics

The results are expressed as the means ±SEM. Changes in sleep architecture, SWA, body temperature, locomotor activity, GTT, and ITT were analysed by two-way repeated measures analysis of variance (ANOVA) followed by Student’s *t* test. The mean duration of sleep, episode number of sleep, and biochemical substance measures were analysed by Student’s *t* test. Drug treatment data (H1RA/BZD) were analysed using one-way ANOVA followed by Tukey’s *post hoc* test, or two-way repeated ANOVA followed by Student’s *t-*test. The number of must cells was compared by Mann-Whitney U test. Cromolyn treatment data were analysed using two-way repeated ANOVA followed by paired *t*-test. P < 0.05 was assumed to indicate statistical significance.

## Electronic supplementary material

Supplementary Information

## References

[1] Chikahisa S (2013). Histamine from brain resident MAST cells promotes wakefulness and modulates behavioral states. PLoS One..

[2] Galli SJ, Grimbaldeston M, Tsai M (2008). Immunomodulatory mast cells: negative, as well as positive, regulators of immunity. Nat Rev Immunol..

[3] Kalesnikoff J, Galli SJ (2008). New developments in mast cell biology. Nat Immunol..

[4] Marshall JS (2004). Mast-cell responses to pathogens. Nat Rev Immunol..

[5] Florenzano F, Bentivoglio M (2000). Degranulation, density, and distribution of mast cells in the rat thalamus: a light and electron microscopic study in basal conditions and after intracerebroventricular administration of nerve growth factor. J Comp Neurol..

[6] Hendrix S (2006). The majority of brain mast cells in B10.PL mice is present in the hippocampal formation. Neurosci Lett..

[7] Mattila OS (2011). Cerebral mast cells mediate blood-brain barrier disruption in acute experimental ischemic stroke through perivascular gelatinase activation. Stroke..

[8] Silverman AJ, Sutherland AK, Wilhelm M, Silver R (2000). Mast cells migrate from blood to brain. J Neurosci..

[9] Cirulli F, Pistillo L, de Acetis L, Alleva E, Aloe L (1998). Increased number of mast cells in the central nervous system of adult male mice following chronic subordination stress. Brain Behav Immun..

[10] Bugajski AJ, Chlap Z, Gadek M, Bugajski J (1994). Effect of isolation stress on brain mast cells and brain histamine levels in rats. Agents Actions..

[11] Chaldakov GN (2001). NGF, BDNF, leptin, and mast cells in human coronary atherosclerosis and metabolic syndrome. Arch Physiol Biochem..

[12] El-Haggar SM, Farrag WF, Kotkata FA (2015). Effect of ketotifen in obese patients with type 2 diabetes mellitus. J Diabetes Complications..

[13] Zhou Y (2015). Leptin Deficiency Shifts Mast Cells toward Anti-Inflammatory Actions and Protects Mice from Obesity and Diabetes by Polarizing M2 Macrophages. Cell Metab..

[14] Liu J (2009). Genetic deficiency and pharmacological stabilization of mast cells reduce diet-induced obesity and diabetes in mice. Nat Med..

[15] Yamatodani A, Maeyama K, Watanabe T, Wada H, Kitamura Y (1982). Tissue distribution of histamine in a mutant mouse deficient in mast cells: clear evidence for the presence of non-mast-cell histamine. Biochem Pharmacol..

[16] Stamatakis KA, Punjabi NM (2010). Effects of sleep fragmentation on glucose metabolism in normal subjects. Chest..

[17] Tasali E, Leproult R, Ehrmann DA, Van Cauter E (2008). Slow-wave sleep and the risk of type 2 diabetes in humans. Proc Natl Acad Sci USA.

[18] Anvari E, Wang X, Sandler S, Welsh N (2015). The H1-receptor antagonist cetirizine ameliorates high-fat diet-induced glucose intolerance in male C57BL/6 mice, but not diabetes outcome in female non-obese diabetic (NOD) mice. Ups J Med Sci..

[19] Theoharides TC (2004). Mast cells as targets of corticotropin-releasing factor and related peptides. Trends Pharmacol Sci..

[20] Theoharides TC (1995). Stress-induced intracranial mast cell degranulation: a corticotropin-releasing hormone-mediated effect. Endocrinology..

[21] Esler MD, Hasking GJ, Willett IR, Leonard PW, Jennings GL (1985). Noradrenaline release and sympathetic nervous system activity. J Hypertens..

[22] Chikahisa S (2008). Bezafibrate, a peroxisome proliferator-activated receptors agonist, decreases body temperature and enhances electroencephalogram delta-oscillation during sleep in mice. Endocrinology..

[23] Chikahisa S, Fujiki N, Kitaoka K, Shimizu N, Sei H (2009). Central AMPK contributes to sleep homeostasis in mice. Neuropharmacology..

