# Supplementary material for: Mast cell involvement in glucose tolerance impairment caused by chronic mild stress with sleep disturbance
Source: Sci Rep. 2017 Oct 20;7:13640. doi: 10.1038/s41598-017-14162-w (PMC5651881; doi:10.1038/s41598-017-14162-w)
Supplement: Supplementary file 1 — Supplementary Information [file 41598_2017_14162_MOESM1_ESM.pdf]

Mast cell involvement in glucose tolerance impairment caused by chronic mild stress with sleep disturbance

Sachiko Chikahisa[1], Saki Harada[1,2], Noriyuki Shimizu[1], Tetsuya Shiuchi[1], Airi Otsuka[1], Seiji Nishino[3], Hiroyoshi Séi[1]*

[1] *Department of Integrative Physiology, Institute of Biomedical Sciences, Tokushima University Graduate School, Tokushima, 770-8503, Japan*

[2] *Student lab, Tokushima University Faculty of Medicine, Tokushima 770-8503 Japan*

[3.] *Sleep & Circadian Neurobiology Laboratory, Stanford University School of Medicine, Palo Alto, CA, United States*

* Corresponding author: Hiroyoshi Séi, M.D., Ph.D.
Department of Integrative Physiology, Institute of Biomedical Sciences, Tokushima University Graduate School, 3-18-15 Kuramoto-cho, Tokushima, 770-8503, Japan
E-mail: sei@tokushima-u.ac.jp, Tel: +81 88 633 7057, Fax: +81 88 633 9251

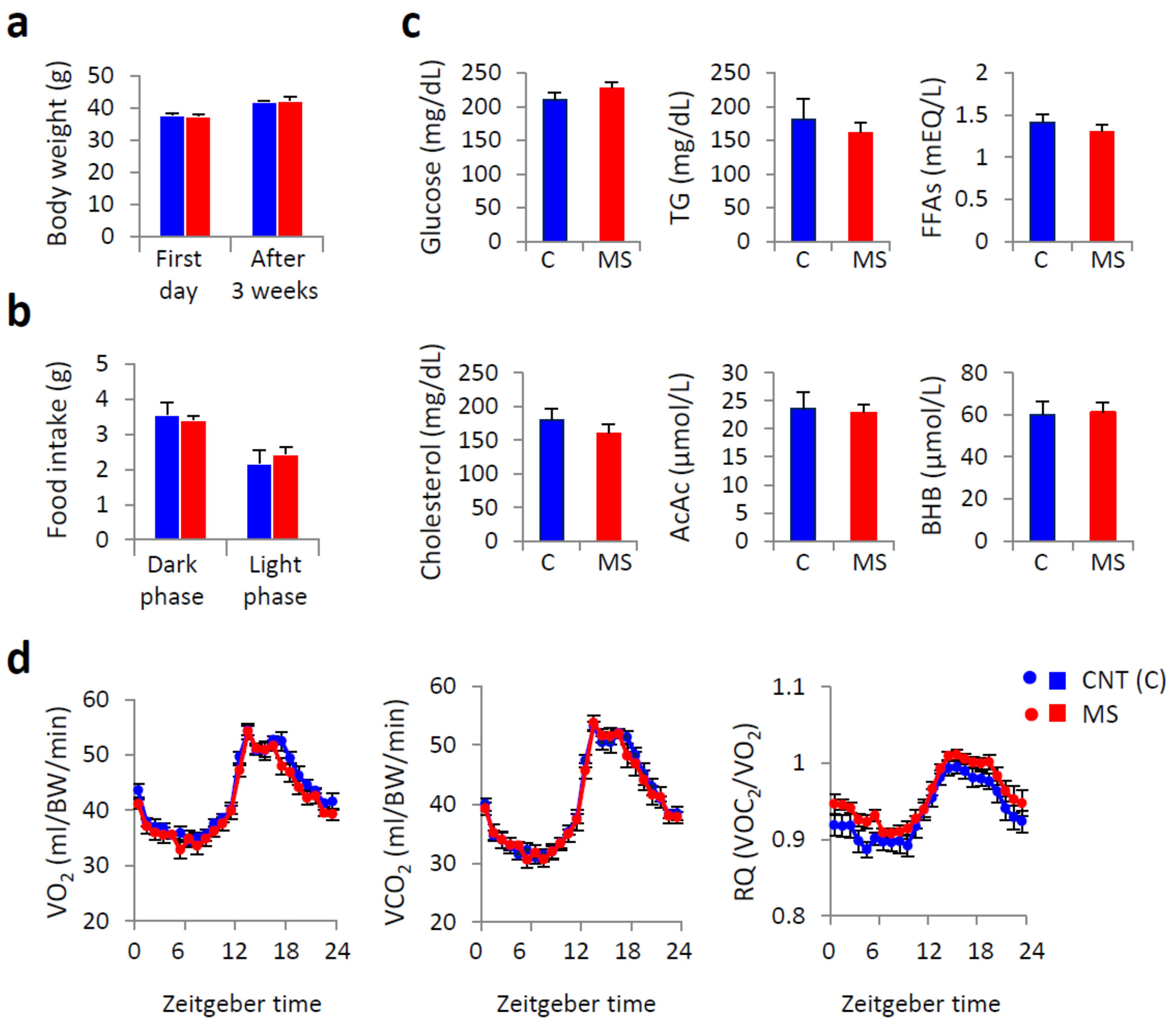

Supplemental figure S1. Effects of chronic mild stress (MS) via rearing on wire net on the metabolic state of mice. (a) Body weight on the first day and 3 weeks after rearing on a wire net. (b) Food intake in the dark and light phase in control (CNT) and MS mice. (c) The plasma levels of glucose, triglycerides (TG), free fatty acids (FFAs), cholesterol, acetoacetate (AcAc) and β-hydroxybutyrate (BHB). (d) Measurement of O_2 consumption (VO_2)(left panel), CO_2 production (VCO_2)(middle panel), and respiratory quotient (RQ) (right panel) in CNT and MS mice. All data are expressed as the means ± SEM (n = 6-7/group).

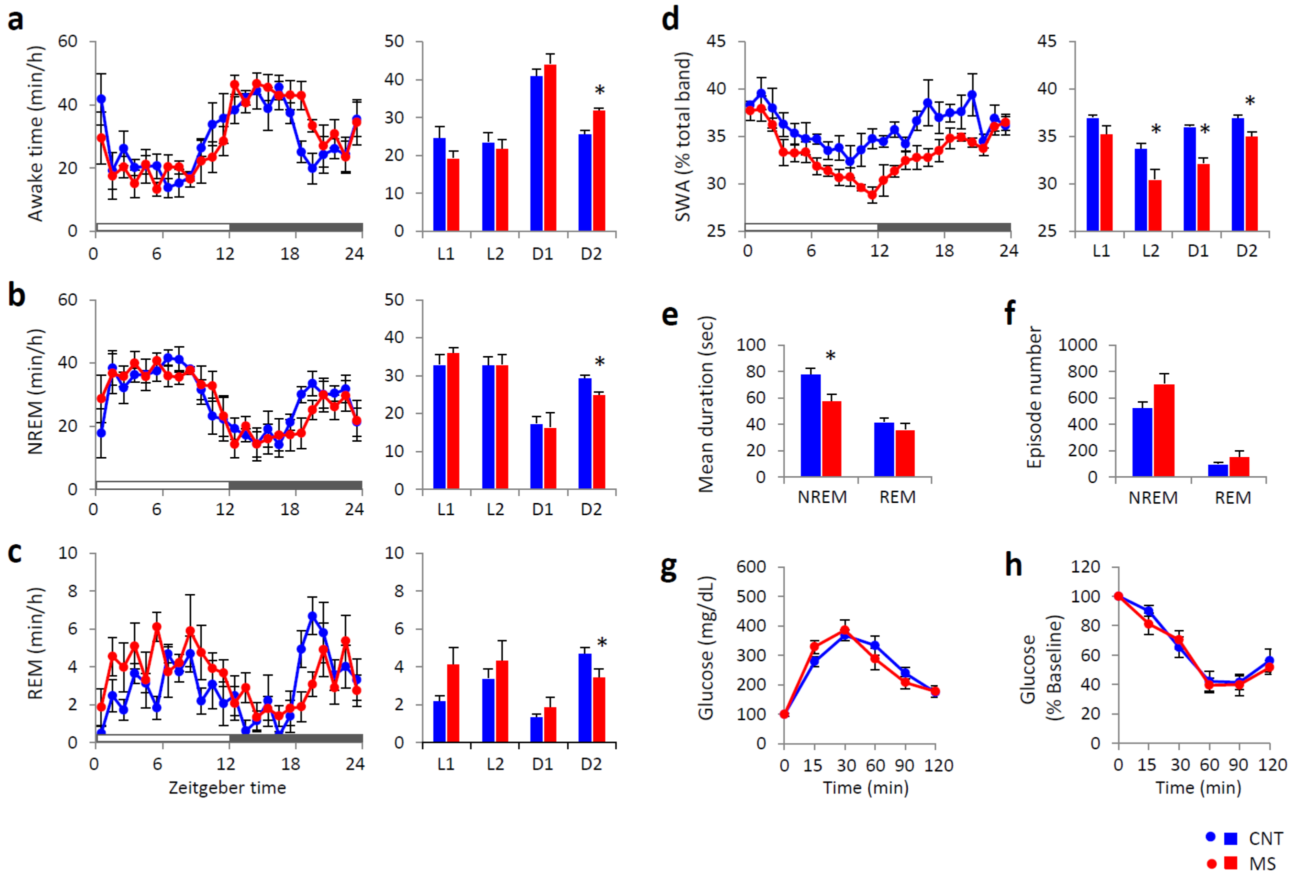

Supplemental figure S2. Rearing on a wire net for 1 week disturbed sleep, but did not impair glucose tolerance. Hourly time course (left panels) and 6-hour bins (right panels; ZT0-6 (L1), ZT6-12 (L2), ZT12-18 (D1) and ZT18-24 (D2)) for wakefulness (a), non-rapid eye movement (NREM) sleep (b) and rapid eye movement (REM) sleep (c), and slow-wave activity (SWA) in NREM sleep (d) after rearing on a wire net for 1 week. Mean duration of bouts (e) and episode number (f) of NREM and REM sleep across 24 hours. An intraperitoneal glucose tolerance test (GTT)(g) and an insulin tolerance test (ITT)(h) were performed in control (CNT) and chronic mild stress (MS) mice. All data are expressed as the means ± SEM (n = 5/group). *$p < 0.05$, CNT versus MS mice.

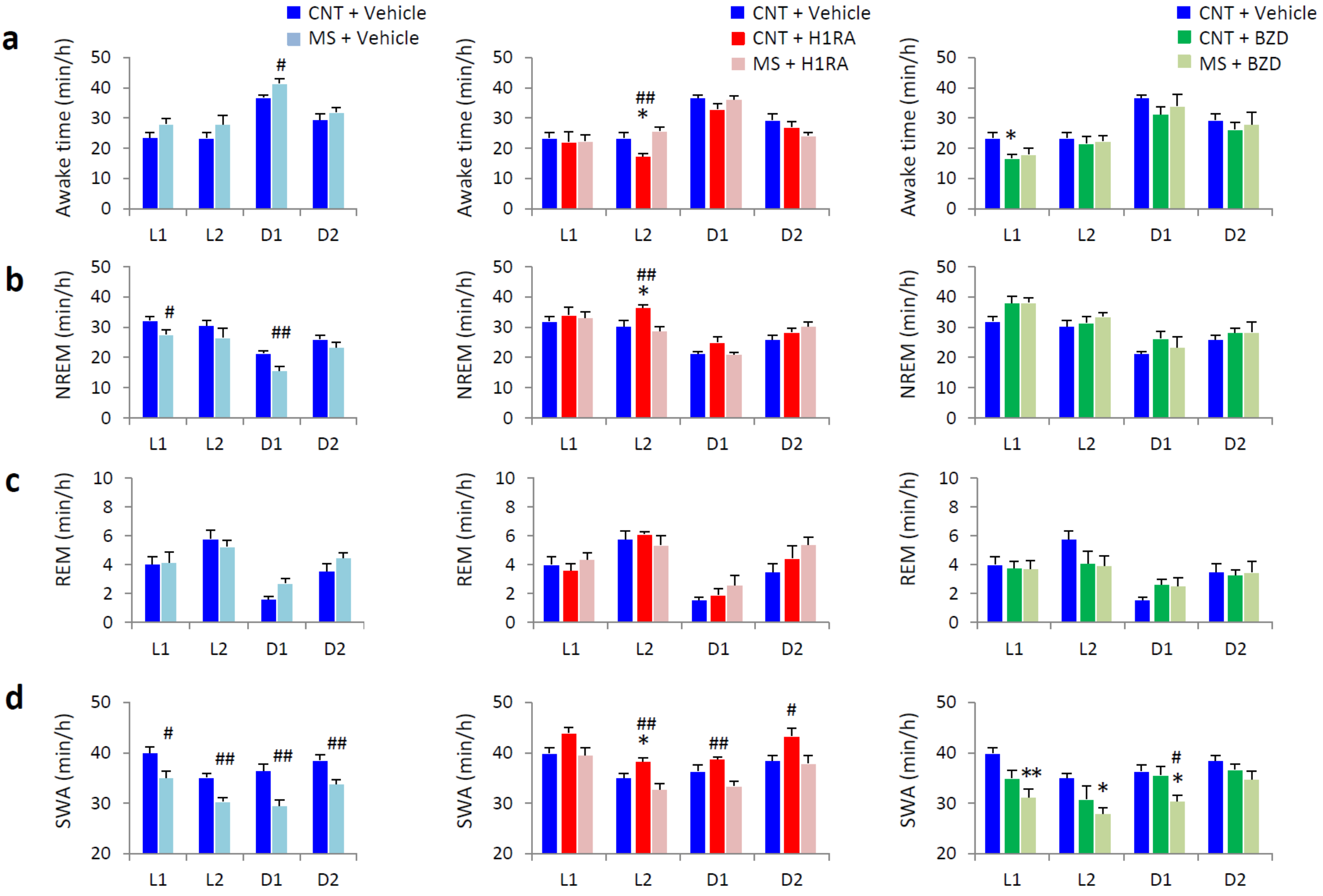

Supplemental figure S3. Sleep changes in mice injected with vehicle, histamine H1 receptor antagonist (H1RA), or benzodiazepine (BZD); a comparison between control (CNT) and chronic mild stress (MS) mice. Six-hour bins (ZT0-6 (L1), ZT6-12 (L2), ZT12-18 (D1) and ZT18-24 (D2)) for wakefulness (a), non-rapid eye movement (NREM) sleep (b) and rapid eye movement (REM) sleep (c), and slow-wave activity (SWA) in NREM sleep (d). Sleep data of vehicle-treated CNT mice (rich blue bars) are duplicated in H1RA/BZD data. All data are expressed as the means ± SEM (n = 6/group). *$p < 0.05$, * * $p < 0.01$, vs vehicle-treated CNT. $^{\#}p < 0.05$, $^{\#\#}p < 0.01$, CNT vs MS in vehicle, H1RA and BZD treatment respectively. (Data in Figure 5 was re-arranged in this figure.)

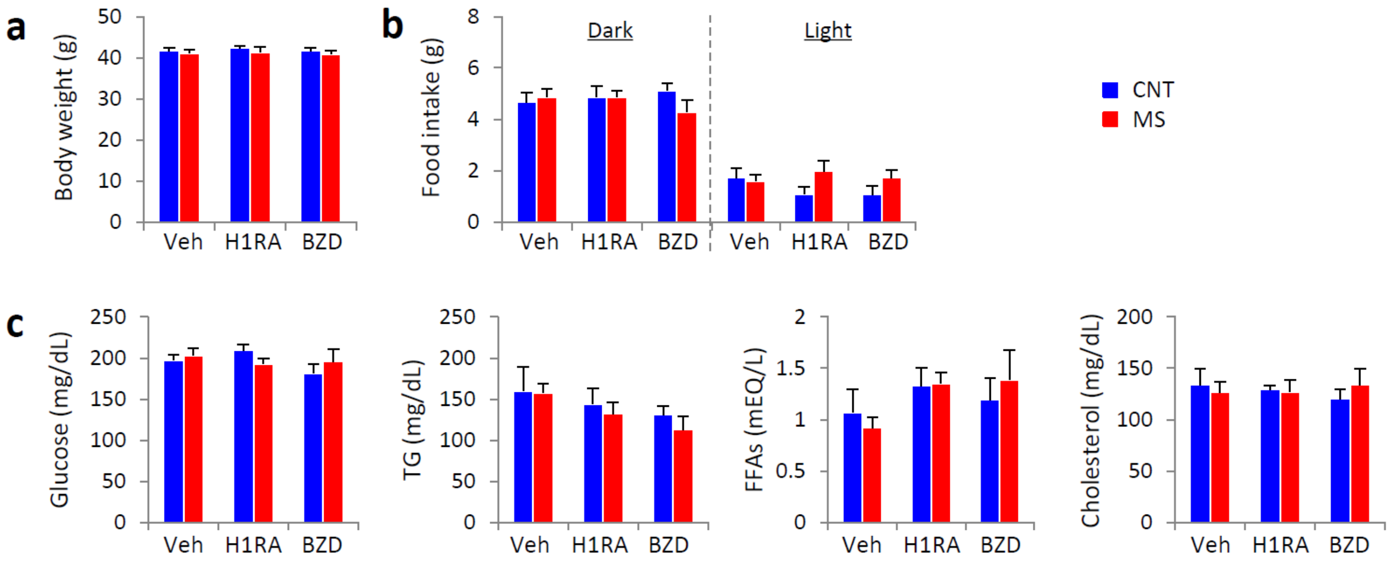

Supplemental figure S4. Two kinds of sleep-inducing agents did not affect the metabolic state of control (CNT) **or chronic mild stress (MS) mice.** Body weight (a) and food intake in the dark and light phase (b) in mice injected with vehicle, histamine H1 receptor antagonist (H1RA), or benzodiazepine (BZD). (c) The plasma levels of glucose, triglycerides (TG), free fatty acids (FFAs), and cholesterol in CNT and MS mice. All data are expressed as the means ± SEM (n = 5/group).

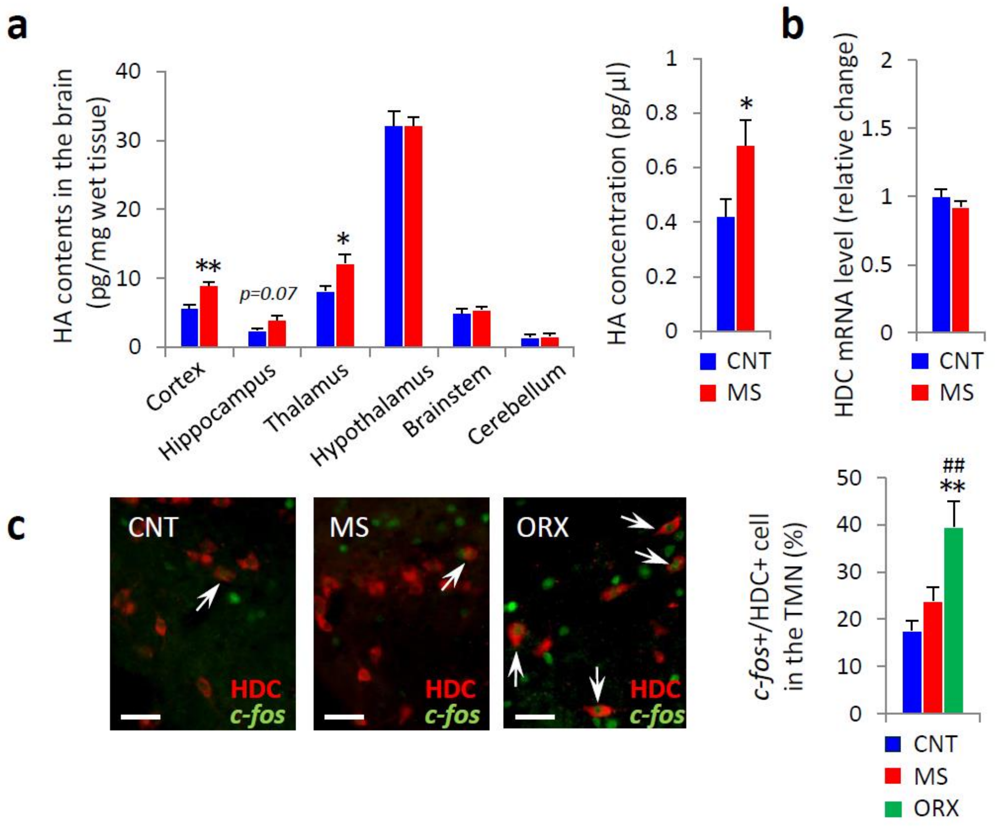

Supplemental figure S5. Increased histamine (HA) levels in chronic mild stress (MS) mice. HA contents in each region of the brain (left panel) and HA release (measured in the lateral ventricle via microdialysis)(right panel) in control (CNT) and MS mice (a). Histidine decarboxylase (HDC) mRNA expression in the hypothalamus (b). Coronal section through mouse TMN region showing c-fos-positive histaminergic neurons (c). Immunoreactivity for HDC (red) and c-fos (green) are localized in the TMN of control, MS, and orexin B-injected (ORX, 1 nmol, i.c.v.) mice (left figures). The merged image shows c-fos-positive HDC-immunoreactive cell bodies (shown by arrows). Cell numbers of c-fos-positive HDC cells are expressed as a percentage of the total HDC-immunoreactive cell numbers (right panel). Scale bar, 100 μm. Blue bars indicate control mice, red bars indicate MS mice, and green bars indicate orexin B-injected mice. All data are expressed as the means ± SEM (n = 6-8/group). $^{*}p < 0.05$, $^{**}p < 0.01$, versus CNT; $^{\#}p < 0.05$, $^{\#\#}p < 0.01$, versus MS.

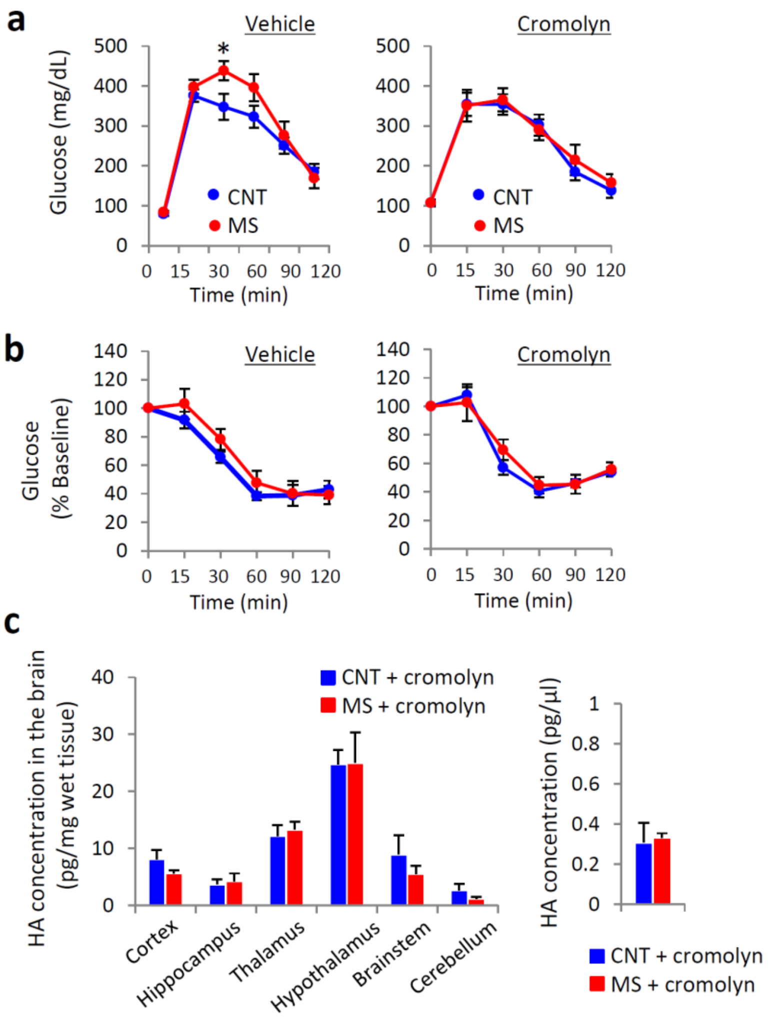

Supplemental figure S6. Inhibition of mast cell degranulation ameliorated the impairment of glucose tolerance in chronic mild stress (MS) mice. An intraperitoneal glucose tolerance test (GTT)(a) and an insulin tolerance test (ITT)(b) were performed in control (CNT) and MS mice injected with vehicle (left panels) or cromolyn (30 μg, i.c.v.)(right panels), a mast cell degranulation blocker. Histamine (HA) contents in each region of the brain (left panel) and HA release (measured in the lateral ventricle via microdialysis)(right panel) in cromolyn-treated CNT and cromolyn-treated MS mice (c). All data are expressed as the means ± SEM (a, b: n = 4-6/group, c: n = 7/group). *$p < 0.05$, CNT versus MS mice.

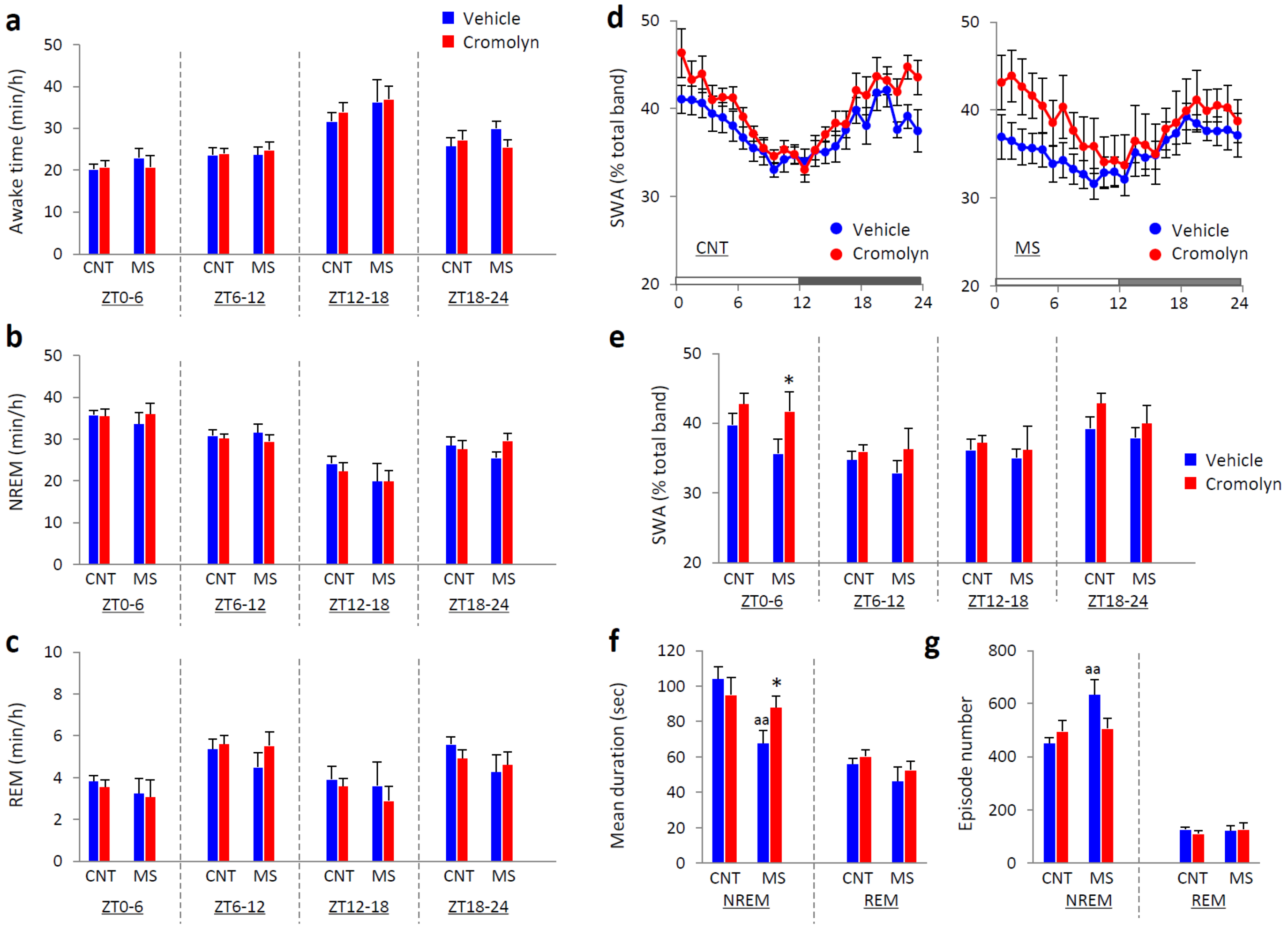

Supplemental figure S7. Inhibition of mast cell degranulation enhanced slow-wave activity (SWA) during non-rapid eye movement (NREM) sleep in chronic mild stress (MS) mice. Time course for 6-hour bins (ZT0-6, ZT6-12, ZT12-18 and ZT18-24) of wakefulness (a), NREM sleep (b), rapid eye movement (REM) sleep (c), and SWA in NREM sleep (e) in control (CNT) and MS mice injected with vehicle or cromolyn (30 μg, i.c.v.). Hourly time course for SWA during NREM sleep (d) and mean duration of bouts (f)/episode number (g) of NREM and REM sleep over 24 hours after drug injection. All data are expressed as the means ± SEM (n = 6-7/group). *$p < 0.05$, vehicle versus cromolyn. $^{aa}p < 0.01$, vehicle-treated CNT versus vehicle-treated MS.

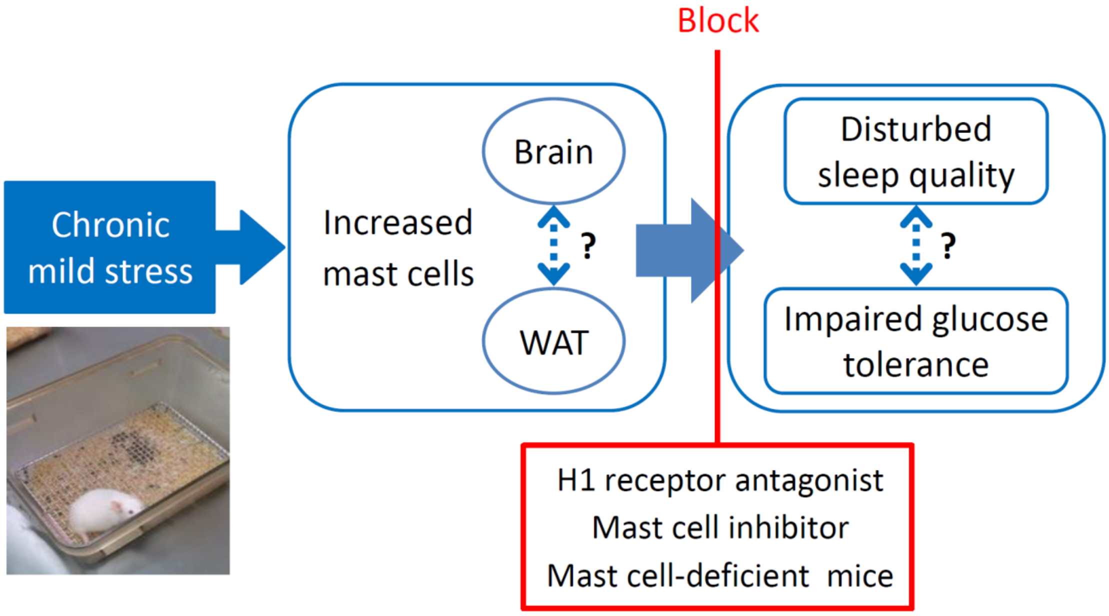

Supplemental figure S8. Schematic drawing illustrating our model of the effect of chronic mild stress on glucose and sleep homeostasis. Chronic mild stress via rearing on a wire net for 3 weeks disturbed sleep quantity and impaired glucose tolerance. This chronic mild stress also increased mast cells in the brain and adipose tissue. Inhibition of mast cell function ameliorated the impairment in both glucose tolerance and sleep. Mast cells may contribute to sleep disturbance-induced dysregulation of glucose homeostasis.
